# Patient-reported, psychosocial and health economic outcomes in mild to moderate Friedreich's ataxia: baseline results of the PROFA study

**DOI:** 10.1016/j.lanepe.2025.101552

**Published:** 2025-12-11

**Authors:** Marcus Grobe-Einsler, Stéphanie Borel, Maresa Buchholz, Sabrina Sayah, Rania Hilab, Lucie Pierron, Audrey Iskandar, Brittany Humphries, Claire Ewenczyk, Anna Heinzmann, Mariana Atencio, Katrin Feldmann, Vivian Maas, Jennifer Faber, Sylvia Boesch, Elisabetta Indelicato, Kathrin Reetz, Jörg B. Schulz, Almut T. Bischoff, Thomas Klopstock, Ludger Schöls, Martina Minnerop, Dagmar Timmann, Elin H. Davies, Thomas Klockgether, Alexandra Durr, Feng Xie, Bernhard Michalowsky

**Affiliations:** a German Center for Neurodegenerative Diseases, Bonn, Germany; b Center for Neurology, Department of Parkinson's Disease, Sleep and Movement Disorders, University Hospital Bonn, Bonn, Germany; c Department of Neurology, Center for Movement Disorders and Neuromodulation, Medical Faculty, Heinrich-Heine University, Düsseldorf, Germany; d Sorbonne Université, Paris Brain Institute (ICM - Institut du Cerveau), INSERM, CNRS, Assistance Publique-Hôpitaux de Paris (AP-HP), Paris, France; e German Center for Neurodegenerative Diseases (DZNE), Site Rostock/Greifswald, Patient-reported Outcomes & Health Economics Research, Greifswald, Germany; f Department of Health Research Methods, Evidence and Impact, McMaster University, Hamilton, Ontario, Canada; g Department of Neuroradiology, University Hospital Bonn, Germany; h Centre for Rare Movement disorders Innsbruck, Department of Neurology, Medical University Innsbruck, Innsbruck, Austria; i Department of Neurology, RWTH Aachen University, Aachen, Germany; j Friedrich-baur-Institute at the Department of Neurology, LMU University Hospital, Ludwig-Maximilian-Universität (LMU) Munich, Germany; k German Center for Neurodegenerative Diseases, Munich, Germany; l Munich Cluster for Systems Neurology (SyNergy), Munich, Germany; m German Center for Neurodegenerative Diseases (DZNE), Site Tübingen, Germany; n Department of Neurology and Hertie Institute for Clinical Brain Research, University of Tuebingen, Germany; o Institute of Neuroscience and Medicine (INM-1), Research Centre Jülich, Jülich, Germany; p Institute of Clinical Neuroscience and Medical Psychology, Medical Faculty & University Hospital Düsseldorf, Heinrich Heine University Düsseldorf, Düsseldorf, Germany; q Department of Neurology and Center for Translational Neuro- and Behavioral Sciences (C-TNBS), Essen University Hospital, University of Duisburg-Essen, Essen, Germany; r Aparito Ltd a Wholly Owned Subsidiary of Eli Lilly and Company, 11-12 Gwenfro, Wrexham Technology Park, Wrexham, Wales, United Kingdom

**Keywords:** PROFA, Friedreich, Ataxia, FA, Health, Economic, Psychosocial

## Abstract

**Background:**

Friedreich ataxia (FA) is the most common autosomal recessive ataxia. Little attention has been paid to FA's impact on patient-reported, psychosocial, and health-economic outcomes. This study aimed to report these outcomes across FA's disability stages 1–5.

**Methods:**

We assessed patients in Germany, France, and Austria as part of the PROFA study, a European multicenter observational study. The protocol included a study center visit followed by a remote mobile assessment capturing ataxia severity (SARA), daily living deficits (FARS-ADL), cognitive and affective impairments (CCAS), health-related quality of life (HRQoL: PROM-Ataxia short-form, EQ-5D-5L), mental well-being (WEMWBS), communication disabilities (COMATAX), and healthcare and informal care utilization. FARS disability stages were used to demonstrate outcomes with effect size measures (Eta-Squared, Cramér's V). Multivariate regression models evaluated associations between z-standardized outcomes and disability stages.

**Findings:**

One hundred one patients (mean [SD]: age 35.0 [11.5]; GAA-repeat size 657 [299]; 50.5% women) were included. Activities of daily living, HRQoL, communication disabilities, and informal care utilization worsened significantly across disability stages with moderate to high effect sizes. Cognitive-affective impairments and mental well-being showed significant associations with small effect sizes. Twenty-three patients (33.3%) received formal care, while 40 (58.0%) received informal care (mean 12.2 h/week). Omaveloxolone was used by 33 patients (32.7%). Annual healthcare costs excluding Omaveloxolone were €13,620 (payer) and €32,679 (societal perspective, including informal care and productivity losses).

**Interpretation:**

The results emphasize the multidimensional patient, societal, and economic burden of FA and the need for comprehensive care addressing physical, mental, and psychosocial health.

**Funding:**

European Joint Programme on Rare Diseases (EJP RD).

**Research in context**

**Evidence before this study**

Friedreich Ataxia (FA) is a rare, autosomal recessive, multisystemic disorder. Core symptoms include ataxia and sensory loss, which manifest as loss of balance, impaired coordination, and difficulty with speech. We searched PubMed using English search terms [(“Friedreich Ataxia”) AND (“patient-reported outcomes” OR “quality of life” OR “psychosocial” OR “communication” OR “utilization” OR “costs”)] for studies published until August 2025, identifying few studies showing that FA significantly impairs health-related quality of life, particularly in the physical domain. Evidence on FAs' impact on psychosocial health, communication disabilities, and economic burden remains limited and fragmented, with studies demonstrating increased healthcare utilization and informal care needs in advanced FA stages. However, comprehensive analyses on patient-reported, psychosocial, and economic outcomes across defined disability stages of FA are lacking.

**Added value of this study**

The PROFA study is the first large-scale, multicenter study that comprehensively evaluates patient-reported outcomes, psychosocial health, and economic burden across FAs' disability stages. The study includes patients from Germany, France, and Austria, combining clinical assessments at the respective study centers with remote app-based data collection at home. As the disability increased, patients experienced a worsening quality of life, reduced ability to manage everyday tasks, and increased communication difficulties. Patients' mental well-being remained relatively low and stable across disability stages. Informal care and societal healthcare costs rose sharply, especially in patients with severe disability.

**Implications of all the available evidence**

Our findings establish clear associations between the FA disability stages and the multidimensional disease burden, underscoring the complex and progressive nature of FA and its wide-ranging effects on individuals, their families, and the healthcare system. This emphasizes the importance of comprehensive, multidisciplinary person-centred care necessary to improve the daily lives of patients with FA. The high percentage and amount of informal care across disability stages also call for better resource allocation, support for family caregivers, and tailored psychosocial interventions. The results provide a foundation for evaluating the impact of new treatments, such as Omaveloxolone, on patient-reported outcomes and healthcare systems.

## Introduction

Friedreich Ataxia (FA) is the most common hereditary form of ataxia, with a prevalence of approximately 2–4 cases per 100,000 people.^1^ This autosomal recessive disease is caused by GAA repeat expansions and or point mutations in the *FXN* gene, resulting in reduced tissue levels of functional frataxin protein.^2^ Neurodegeneration in FA primarily affects the spinal cord, the cerebellum, and the peripheral nervous system. The resulting core clinical features include ataxia and sensory loss, which manifest as loss of balance, impaired coordination, and difficulty with speech. Involvement of additional organ systems results in cardiac complications, such as cardiomyopathy and arrhythmia, diabetes, skeletal deformities, as well as hearing and visual impairment. The first symptoms usually appear around puberty and progress over decades, with early loss of ambulation and a reduced life expectancy of less than 40 years.^1^

There are only a few studies on health-related quality of life (HRQoL) in FA. These studies have shown a high impact of FA on patients' HRQoL, particularly in the physical domains.^3^, ^4^, ^5^, ^6^, ^7^, ^8^, ^9^ Little attention has been paid to communication disabilities. Their impact on psychosocial health has not been measured so far. Speech and language disorders are prominent signs in FA and often evolve into unintelligible speech, which dramatically impairs communication with others and the expression of needs or emotions.^10^^,^^11^ Furthermore, hearing impairment can exacerbate severe communication problems, especially in noisy environments (auditory neuropathy).^12^ Both disabilities are associated with decreased HRQoL, exacerbation of social isolation, and alterations in daily living activities, increasing the risk of developing affective disorders, such as depression. Mild cognitive impairment combined with high persistence and low self-transcendence, which is summarized as a cerebellar cognitive affective syndrome, has also been reported among FA patients, additionally affecting patients' HRQoL and social life.^13^

The growing functional decline in patients imposes a significant economic burden on healthcare utilization, reducing the employability of patients and their informal caregivers. Only a few studies have evaluated the economic consequences of FA. Polek and colleagues revealed that healthcare utilization is higher in advanced disease stages, with paid home care being the main cost driver.^14^ However, the burden of FA also falls on society. Family caregivers, in most cases parents, provide up to 51 h of informal care each week, and 25%–30% of patients are unemployed due to FA.^15^ Such tremendous productivity losses contribute to the disease's indirect costs and underline the complex implications of FA disease on patients, caregivers, and healthcare systems.^7^

Studies showing the impact of FAs on HRQoL, well-being, psychosocial health, and economic outcomes across disease severity are essential to understanding the disease's impact on patients' daily lives, its interplay with informal caregivers, and the healthcare system. This is especially important because of the recent approval of Omaveloxolone, a nuclear factor erythroid 2-related factor 2 (Nrf2) activator. Given that previous studies are based on cross-sectional data and small sample sizes, it is difficult to generalize the limited data available to larger cohorts. Studies showing the impact of FA on HRQoL, well-being, psychosocial health, and economic outcomes across disease severity are essential to understanding the disease's impact on patients' daily lives, its interplay with informal caregivers, and the healthcare system. Given that previous studies are based on cross-sectional data and small sample sizes, it is difficult to generalize the limited data available to larger cohorts.

Therefore, this study aimed not only to describe clinical aspects of FA based on the PROFA baseline data but also to demonstrate the patient-reported, psychosocial, and economic impact of FA across FA disability stages in a large cohort of FA patients across Europe. We hypothesize that patient-relevant outcomes significantly worsened across stages.

## Methods

### Study design

The PROFA study (“Patient-reported, health economic and psychosocial outcomes in patients with Friedreich ataxia”, NCT05943002) is a prospective observational study in FA, recruiting from six European study centers (Germany, France, and Austria).^16^ The study design is outlined in Supplementary Figure S1. The inclusion criteria were a genetically confirmed diagnosis of FA, age ≥12 years, access to a smart device and a Wi-Fi connection at home, and a score on the Scale for Assessment and Rating of Ataxia (SARA) of <30 points.^17^ The latter was selected to ensure patients would be capable of handling a smart device. After a baseline assessment at the study center, patients completed a series of assessments via a mobile health app (ATOM5 by Aparito) over a six-month period at home. The complete study protocol has been published previously.^16^ Due to ongoing longitudinal assessments, this analysis includes only the baseline assessments.

### Data assessments

A detailed description of each outcome measure is provided in Supplementary Table S1. Baseline assessment at the study centers included documentation of medical history, GAA trinucleotide repeat size, demographics, and education level (International Standard Classification of Education). Measures of disease severity included the SARA, FARS disability stages and Inventory of Non-Ataxia Signs (INAS), and hearing loss assessed by HearWHO.^17^, ^18^, ^19^ Cognitive function was assessed using the Cerebellar Cognitive Affective Syndrome Scale (CCAS),^20^ and Activities of Daily Living with the relevant section of the Friedreich Ataxia Rating Scale (FARS-ADL).^19^ Additionally, patients remotely completed the following questionnaires via the app at home within two weeks after the study center visit: Health-related quality of life measures, including the ataxia-specific short version of the PROM-Ataxia and the generic EQ-5D-5L and EQ-5D-Y-5L for participants aged 12–16,^21^^,^^22^ psychological well-being assessed by the Warwick–Edinburgh Mental Well-being Scale (WEMWBS),^23^ and psychosocial impact of communication disabilities assessed by the newly developed COMATAX, a patient reported outcome measure covering the domains speech, hearing, language, emotions, the connection between communication and fatigue, and the psychological implications of communication difficulties with 17 items that are rated on a 5-point Likert scale (0–4) (see detailes description in Supplementary Table S1).

Healthcare service utilization and informal care were also retrospectively assessed via the app two months after the study center visit, using the Questionnaire for Health-Related Resource Use (FIMA) and Resource Utilization (RUD) questionnaires for the last two months.^24^^,^^25^ Healthcare utilization includes physician visits, in-hospital treatments, medication, therapies, medical aids and formal home care and institutionalized care. While professional care services provide formal and institutionalized care, informal care captures caregiver support (non-professional) for activities of daily living and instrumental activities of daily living, as well as caregivers' short-term and long-term productivity losses.^25^

Healthcare costs, informal care provision and caregiver productivity losses were monetarized using standardized unit costs and the opportunity cost approach in 2024 values (€), as described in Supplementary Table S2. To prevent overlap, informal care time and caregiver productivity losses were accounted for separately, ensuring that no double-counting occurred in the societal cost estimates.

Costs were calculated from the payer perspective, including medical and formal care only, and from a societal standpoint, including caregiver informal care and productivity losses. Costs for Omaveloxolone (currently ∼327 k€ annually) were excluded from the analysis, as the medication became available during the study period.^26^

### Statistical analyses

Patient characteristics were demonstrated descriptively. The association between repeat length and age of onset was evaluated by Spearman correlation. We used t-tests and Fisher–Exact tests to compare clinical characteristics and outcomes in patients with a typical onset of <20 years (referred to as “typical” onset) and ≥20 years (referred to as “late”) onset.^27^^,^^28^ Among the selected outcomes, the FARS disability stages represent a patient-centric evaluation of functional impairments and were therefore selected as the primary anchor for the following analyses. Patient-reported, psychosocial, and health-economic outcomes were presented descriptively across FARS disability stages (and SARA categories as a sensitivity analysis; see Supplementary Material). Effect sizes across FARS disability stages were measured using Eta Squared (η^2^) and Cramér V, indicating small (η^2^ ≥ 0.01; V ≥ 0.1), medium (η^2^ ≥ 0.06; V ≥ 0.3), and large (η^2^ ≥ 0.14; V ≥ 0.5) effects.

We performed multivariate linear (patient-reported outcomes) and generalized estimating equation models (economic outcomes; gamma family with log link) to assess the associations between the patient-reported, psychosocial and health-economic outcome variables as a dependent variable and the FARS disability stage as the predictor of interest. To enhance comparability across models, all outcome variables were also z-standardized and adjusted for age, sex, disease duration and education. We used random effects for the respective study center to account for clustering. We calculated the coefficient of determination (R^2^) to represent the proportion of variance in standardized outcomes explained by the disability stages, with higher values indicating stronger associations. Additionally, we calculated the gain in R^2^ to quantify the additional variance explained when disability stages are included in the model. Results were demonstrated using margin plots. All statistical analyses were conducted as complete case analyses. Missing values are presented in. Statistical analysis were performed using STATA 16.

### Ethics approval

The study was approved by the Ethics Committee of the University of Medicine Greifswald (BB096/22a, October 26 2022) and all local ethics committees of participating study sites (RWTH Aachen, Faculty of Medicine: 22-014; University of Bonn: 440/22; Innsbruck: Medical University of Innsbruck: 1379/2022, Munich, Medical Faculty: 22–1095; Paris: Comité de Protection des Personnes Est III: 2023-A00315-40; Tübingen, University Tübingen Faculty of Medicine: 672/2022BO2), and was conducted according to the Declaration of Helsinki. All participants and parents of participants under 18 years provided written informed consent.

### Role of the funding source

The funders did not influence the study design, data collection, data analysis, interpretation, or writing of the manuscript.

## Results

### Patient characteristics

**Fig. 1:**
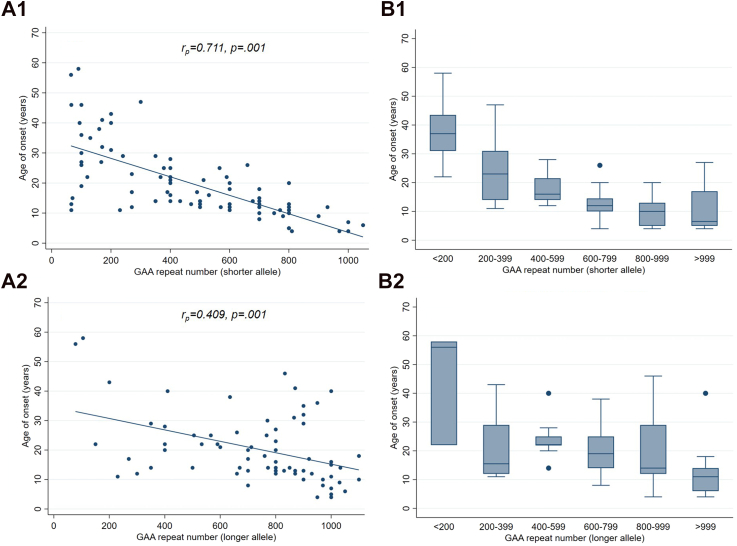
Genetic background (FXN GAA repeat number of shorter and longer allele) and age of onset. (A) Age of onset plotted against FXN GAA repeat number of shorter (A.1) and longer (A.2) allele. (B) Box plots of age of onset plotted against FXN GAA repeat number of shorter (B.1) and longer (B.2) alleles.

Between June 1st, 2023, and October 31st, 2024, 101 FA patients (mean [SD] age, 35.0 [11.5] years; 51 [50.5%] women) participated in this study (Supplementary Table S4). The mean ataxia severity was 17.5 (±5.9) SARA points with a mean FARS disability stage of 4.4 (±1.3). Disability stage 0 and 6 were not present in this cohort. The mean reported age of onset was 19.2 (±11.5) years. The mean shorter/longer allele GAA repeat size was 534 (±289)/812 (±410). There were moderate to strong correlations between age of onset and the shorter (r_p_ = 0.771, p = 0.001) and longer allele GAA repeat size (r_p_ = .409, p = 0.001) (Fig. 1).

Sixty-two patients (61.4%) reported a typical FA onset, whereas 39 (38.6%) reported late onset. The mean disease duration tended to be higher in the late-onset group (14.7 ± 8.45 vs 17.6 ± 8.58 years, p = 0.092). In the typical onset group, patient age was lower (26.6 vs 48.4 years, p = 0.001), fewer patients had children (16.4% vs 66.7%, p = 0.001), but more likely met the criteria of definite CCAS (54.4% vs 39.5%, p = 0.049). For all other outcomes, no significant differences between typical and late-onset were detected.

The socio-demographics of the patients, as well as the outcomes of the entire sample and across typical- and late-onset groups, are presented in Table 1.

**Table 1:** Baseline characteristics across onset group.

	Total cohort (N = 101)^b^	Typical-onset <20 (N = 62)^b^	Late-onset ≥20 (N = 39)^b^	p-value^a^
**Sociodemographic**				
Age, mean (SD)	35.0 (14.8)	26.6 (9.4)	48.4 (11.8)	**0.001**
Sex (female), n (%)	51 (50.5)	32 (51.6)	19 (48.7)	0.839
Having children, n (%)	36 (36.0)	10 (16.4)	26 (66.7)	**0.001**
Education level, mean (SD)	4.9 (1.2)	4.8 (1.3)	5.1 (1.1)	0.200
Employment (yes), n (%)	48 (47.5)	26 (41.9)	22 (56.4)	0.219
**Clinical characteristics**				
Age of onset, mean (SD)	19.2 (11.5)	12.0 (3.76)	30.7 (10.13)	**0.001**
FA duration (years), mean (SD)	15.8 (8.6)	14.7 (8.45)	17.6 (8.58)	0.092
Ataxia severity (SARA), mean (SD)	17.5 (5.9)	18.0 (6.13)	16.8 (5.61)	0.313
FA Disability stage, mean (SD)	4.4 (1.3)	4.4 (1.32)	4.3 (1.21)	0.609
Number of FXN GAA repeats^c^				
Short allele (GAA1), mean (SD)	534 (289)	663 (244)	324 (244)	**0.001**
Long allele (GAA2), mean (SD)	812 (410)	895 (327)	692 (493)	**0.034**
Combined (GAA1&2/2), mean (SD)	657 (299)	773 (269)	497 (267)	**0.001**
INAS, mean (SD)	4.4 (1.6)	4.3 (1.5)	4.4 (1.7)	0.750
CCAS, mean (SD)	91.9 (13.4)	90.5 (12.7)	94.0 (14.4)	0.730
Definite CCAS (score ≥3), n (%)	53 (55.8)	31 (54.4)	15 (39.5)	**0.049**
Hearing problems (yes), n (%)	40 (41.2)	28 (47.5)	12 (31.6)	0.143
**Patient-reported outcomes**				
EQ-5D-5L, mean (SD)	0.68 (0.23)	0.67 (0.25)	0.69 (0.19)	0.658
PROM-Ataxia, mean (SD)	20.3 (8.2)	20.3 (9.10)	20.3 (7.00)	0.968
FARS-ADL, mean (SD)	12.7 (4.8)	12.8 (5.2)	12.4 (4.2)	0.719
WEMWBS, mean (SD)	35.8 (9.1)	34.5 (8.7)	37.5 (9.4)	0.135
COMATAX, mean (SD)	23.2 (12.2)	23.0 (13.9)	23.5 (9.5)	0.831
**Health service utilization**				
Social care access^d^, n (%)	13 (18.8)	7 (15.9)	6 (24.0)	0.524
Hospitalized^c^, n (%)	4 (5.8)	3 (6.8)	1 (4.0)	1.000
Formal care support^c^, n (%)	23 (33.3)	16 (36.4)	7 (28.0)	0.598
Omaveloxolone intake, n (%)	33 (32.7)	25 (40.3)	8 (20.5)	**0.050**
**Informal care**				
Informal care provision^c^, n (%)	40 (58.0)	25 (56.8)	15 (60.0)	0.604
Hours per week, mean (SD)	12.2 (21.5)	14.3 (25.1)	8.5 (12.6)	0.300
Caregiver employment reduction, n (%)	11 (16.4)	8 (18.6)	3 (12.5)	0.734
**Health-care costs in € values**				
Payer perspective, mean (SD)	13,620 (28,113)	12,939 (3419)	14,819 (7253)	0.792
Societal perspective, mean (SD)	32,679 (42,582)	35,288 (44,886)	28,089 (38,644)	0.504

**Abbreviations:** FA, Friedreich Ataxia; SD, standard deviation. CCAS, Cerebellar Cognitive Affective Syndrome Scale; FARS-ADL, Friedreich's Ataxia Rating Scale–Activities of Daily Living Subscale; WEMWBS, Warwick–Edinburgh Mental Well-being Scale; COMATAX, scale for the psychosocial impact of communication disabilities; Hearing.

a t-tests were used for continuous variables, bold numbers indicate a statistically significant difference (p ≤ 0.05).

b Number of missing values are demonstrated in Supplementary Table S3; χ^2^ tests (with Fisher's exact for small cells) for categorical variables.

c 5 patients with a mutation point in our population.

d During the last 2 months.

### Description of outcomes

HRQoL, measured by the EQ-5D-5L and the PROM-Ataxia, was valued at 0.68 (±0.23) and 20.3 (±8.2), respectively. The mental well-being scale (WEMWBS) mean score was 35.8 (±9.1). The communication disability, measured by the COMATAX, was 23.2 (±12.2). Forty patients (42.2%) had possible or likely hearing loss. One-third (33.3%) of the patients received formal care, while nearly two-thirds (61.2%) received informal care, with an average of 12.6 h per week. Omaveloxolone was taken by 32.4% of patients and more frequently in the typical onset group (40.3% vs 20.5%; p = 0.05). Annual healthcare costs (excluding Omaveloxolone) were €13,620 (±28,113) from the payer and €32,679 (±42,582) from the societal perspective, accounting for 58% of the total costs in FA.

### Patient outcomes across disability stages

Table 2 summarizes the cross-sectional outcomes across FA disability stages.

**Table 2:** Outcomes across disability stages and ataxia severity.

	FARS disability stages					p-value^b^	Effect size^c^ (η^2^, Cramér's V)
	1: No disability (n = 5)	2: Minimal disability (n = 30)	3: Mild disability (n = 10)	4: Moderate disability (n = 31)	5: Severe disability (n = 24)		
**Clinical characteristics**							
Age of onset, mean (SD)	14.0 (9.0)	20.6 (13.5)	20.6 (13.2)	21.3 (12.2)	15.6 (5.8)	0.295	0.050 ^small^
Ataxia duration (years), mean (SD)	9.4 (4.3)	12.8 (8.7)	9.8 (4.0)	19.1 (8.3)	19.1 (8.0)	**0.001**	0.194 ^large^
Ataxia severity (SARA), mean (SD)	8.5 (3.5)	13.0 (3.4)	15.1 (3.2)	19.8 (4.1)	23.4 (4.5)	**0.001**	0.589 ^large^
INAS, mean (SD)	2.6 (0.9)	3.8 (1.5)	3.5 (0.7)	5.1 (1.6)	5.0 (1.5)	**0.001**	0.224 ^large^
CCAS, mean (SD)	97.4 (7.4)	95.4 (12.1)	96.4 (11.9)	90.4 (15.5)	85.8 (12.5)	0.063	0.094 ^medium^
Hearing problems^a^, n (%)	0 (0.0)	8 (26.7)	4 (44.4)	11 (36.7)	17 (73.9)	**0.013**	0.316 ^medium^
**Patient-reported outcomes**							
EQ-5D-5L, mean (SD)	0.96 (0.06)	0.84 (0.12)	0.58 (0.31)	0.57 (0.26)	0.59 (0.12)	**0.001**	0.360 ^large^
PROM-Ataxia, mean (SD)	6.2 (3.8)	14.8 (7.4)	20.3 (6.7)	24.6 (6.9)	24.6 (4.4)	**0.001**	0.428 ^large^
FARS-ADL, mean (SD)	6.2 (3.0)	9.2 (3.6)	10.8 (2.3)	14.6 (3.6)	16.7 (3.6)	**0.001**	0.490 ^large^
WEMWBS, mean (SD)	40.0 (7.7)	38.8 (9.3)	30.8 (12.2)	34.7 (7.4)	34.4 (9.1)	0.126	0.086 ^medium^
COMATAX, mean (SD)	8.0 (7.9)	18.5 (11.7)	19.4 (6.9)	27.7 (12.2)	29.1 (10.3)	**0.001**	0.242 ^large^
**Health service utilization**							
Social care access^a^, n (%)	1 (25.0)	3 (15.0)	0 (0.0)	5 (26.3)	3 (18.8)	0.530	0.216 ^small^
Hospitalized^a^, n (%)	0 (0.0)	1 (5.0)	0 (0.0)	1 (5.3)	2 (12.5)	0.714	0.177 ^small^
Formal care support^a^, n (%)	1 (25.0)	2 (10.0)	2 (22.2)	7 (36.8)	11 (68.8)	**0.006**	0.463 ^medium^
Omaveloxolone intake^a^, n (%)	3 (60.0)	8 (26.7)	0 (0.0)	13 (41.9)	9 (37.5)	0.076	0.291 ^small^
**Informal care**							
Informal care provision^a^, n (%)	0 (0.0)	9 (45.0)	6 (66.7)	15 (79.0)	10 (71.4)	**0.020**	0.421 ^medium^
Hours per week, mean (SD)	0.0 (0.0)	5.4 (12.3)	14.1 (25.7)	17.0 (28.9)	20.2 (19.6)	0.199	0.092 ^medium^
Caregiver employment reduction, n (%)	1 (25.0)	3 (15.0)	0 (0.0)	4 (21.1)	3 (21.4)	0.639	0.196 ^small^
**Health-care costs in € values**							
Payer perspective, mean (SD)	4526 (3088)	10,394 (21,424)	4318 (3457)	5702 (8803)	30,820 (46,674)	**0.042**	0.143 ^large^
Societal perspective, mean (SD)	6088 (4429)	18,698 (27,770)	26,134 (38,598)	32,084 (46,813)	58,448 (50,030)	**0.039**	0.146 ^large^

**Abbreviations:** FA, Friedreich's Ataxia; SD, standard deviation; CCAS, Cerebellar Cognitive Affective Syndrome Scale; FARS-ADL, Friedreich's Ataxia Rating Scale–Activities of Daily Living Subscale; WEMWBS, Warwick–Edinburgh Mental Well-being Scale; COMATAX, scale for the psychosocial impact of communication disabilities; INAS, Inventory of Non-Ataxia Signs; SARA, Scale for the Assessment and Rating of Ataxia.

a During the last 2 months;^a^ HearWho test, summarizing possible and likely hearing problems.

b One-way ANOVA (with η^2^ as effect size) were used for continuous variables, Pearson χ^2^ tests (with Cramér's V as effect size) for categorical variables, bold numbers indicate a statistically significant difference (p ≤ 0.05).

c Cramers V were used for percentage values, Eta-square (η^2^) for numeric values.

Ataxia severity (SARA), non-ataxia involvement (INAS) and hearing loss (HearWHO) were significantly higher in patients with higher disability stages, with moderate to high effect sizes. Among the patient-related outcome measures, we found worsening values with rising disability stages for limitations in activities of daily living (FARS-ADL, p < 0.001), HRQoL (EQ-5D-5L, p < 0.001; PROM-Ataxia, p < 0.001), communication disabilities (COMATAX, p < 0.001), but not for mental well-being (WEMWBS, p = 0.126).

Moderate effect sizes were found for the FARS-ADL (V = 0.490) and PROM-Ataxia (V = 0.428) only. The COMATAX yielded nearly moderate effect sizes with V = 0.242, while all other outcomes showed small to negligible effect sizes.

The percentage of patients receiving formal and informal care was higher (with p = 0.006 and p = 0.021, respectively) in patients with higher disability stages. Informal care increased (p = 0.013) from 0 h per week (±0.00) in patients with minor disabilities to 20.2 (±19.6) in severely disabled patients. However, the percentage of caregivers' employment reduction had no tendency (p = 0.639). All healthcare and informal care utilization variables yielded large effect sizes, with values exceeding η^2^ = 0.14.

Healthcare costs from a societal perspective increased across disability stages from €6088 (±4429) in patients with no disability to €58,448 (±50,030) in fully disabled patients confined to a wheelchair. Costs from the payer perspective remained stable from the minimal stage (€44,526, ±3088) to the moderate stage (€5,702, ±8803) and significantly increased in patients with full disability (€30,820, ±46,674). Overall, the increase in costs across ADL stages represented small effect sizes.

### Adjusted effects of disability stages on outcomes

All regression models showed significant associations between the disability stage and the included standardized outcomes, except for costs from the payer's perspective, which showed only a trend toward rising costs with increasing disability stage (p = 0.164). Regression results are summarized in Table 3.

**Table 3:** Association between standardized outcomes and disability stages.

Standardized Outcomes	Estimates (beta coef.)	Standard Error	95%	CI	p-value	R^2^	R^2^ gain
CCAS^a^	−0.30	0.08	−0.47	−0.13	**0.000**	16.7	12.0
EQ-5D-5L^b^	−0.38	0.08	−0.53	−0.22	**0.000**	25.2	18.67
PROM-Ataxia^c^	0.47	0.07	0.32	0.61	**0.000**	40.1	28.5
FARS-ADL^d^	0.54	0.06	0.42	0.67	**0.000**	49.6	38.7
WEMWBS^e^	−0.27	0.09	−0.45	−0.09	**0.003**	14.3	9.7
COMATAX^f^	0.38	0.08	0.21	0.53	**0.000**	28.8	18.2
Hearing Loss^g^	0.61	0.21	0.20	1.03	**0.003**	–	–
Informal care time^h^	0.23	0.10	0.03	0.43	**0.023**	17.2	6.6
Health-care Costs (Payer)^i^	0.14	0.10	−0.05	0.34	0.164	9.4	1.5
Health-care Costs (Societal)^j^	0.27	0.10	0.08	0.46	**0.005**	22.9	10.4

**Abbreviations:** CI, confidence interval; R^2^ (gain), coefficient of determination, indicating the proportion of variance explained by the model, with ‘gain’ referring to the additional variance explained when disability stage was added to the baseline model; CCAS, Cerebellar Cognitive Affective Syndrome Scale; FARS-ADL, Activities of Daily Living Subscale of the Friedreich's Ataxia Rating Scale; WEMWBS, Warwick–Edinburgh Mental Well-being Scale; COMATAX, scale for the psychosocial impact of communication disabilities.

**Footnotes:** Linear (logistic^g^) regression model with random effects for study site (cluster) and adjusted for age, sex, education, disease duration; bold numbers indicate a statistically significant difference (p ≤ 0.05).

a n = 94; 6 clusters, 15.7 observation per cluster, Wald chi^2^(5) = 17.64, p = 0.0034.

b n = 96; 6 clusters, 16.0 observation per cluster, Wald chi2(5) = 14.14, p = 0.0148.

c n = 97; 6 clusters, 16.2 observation per cluster, Wald chi2(5) = 60.87, p = 0.0000.

d n = 100; 6 clusters, 16.7 observation per cluster, Wald chi2(5) = 92.49, p = 0.0000.

e n = 84; 6 clusters, 14.0 observation per cluster, Wald chi2(5) = 13.03, p = 0.0231.

f n = 84; 6 clusters, 14.0 observation per cluster, Wald chi2(5) = 31.47, p = 0.0000.

g n = 96; 6 clusters, 16.0 observation per cluster, Wald chi2(5) = 10.68, p = 0.0582.

h n = 66; 6 clusters, 11.0 observation per cluster, Wald chi2(5) = 15.74, p = 0.0076.

i n = 68; 6 clusters, 11.3 observation per cluster, Wald chi2(5) = 6.40, p = 0.2692.

j n = 68; 6 clusters, 11.3 observation per cluster, Wald chi2(5) = 17.58, p = 0.0035.

Standardized coefficients (ß) and explained variance (R^2^) were highest for the association of the disability stages with limitations in activities of daily living (FARS-ADL: ß = 0.54; R^2^ 49.6%; R^2^ gain 38.7%), followed by the ataxia-specific HRQoL PROM-Ataxia (ß = 0.47; R^2^ 40.1%; R^2^ gain 28.5%), communication disabilities (COMATAX: ß = 0.38; R^2^ 28.8%; R^2^ gain 18.2%), generic HRQoL (EQ-5D-5L: ß = −0.38; R^2^ 25.2%; R^2^ gain 18.7%), and cognitive-affective limitations (CCAS: ß = −0.30; R^2^ 16.7%; R^2^ gain 12.0%). Mental well-being (WEMWBS), informal care time, and costs from a societal perspective were significantly associated with disease severity, but yielded lower standardized coefficients and R^2^ values.

**Fig. 2:**
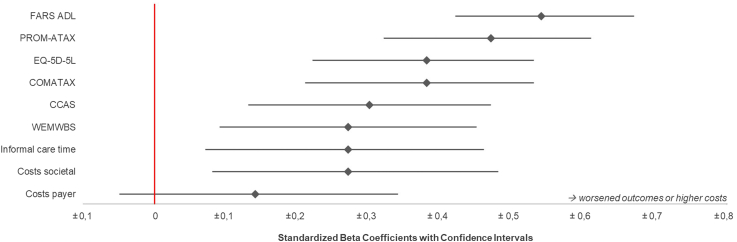
Standardized regression coefficients showing the association between FARS disability stages and outcome measures, adjusted for age, sex, age at disease onset, and education. **Footnotes:** Forrest plots based on a linear regression model with random effects for study site (cluster) and adjusted for age, sex, education, and disease duration. **Abbreviations:** CCAS, Cerebellar Cognitive Affective Syndrome Scale; FARS-ADL, Friedreich's Ataxia Rating Scale–Activities of Daily Living Subscale; WEMWBS, Warwick–Edinburgh Mental Well-being Scale; COMATAX, scale for the psychosocial impact of communication disabilities.

**Fig. 3:**
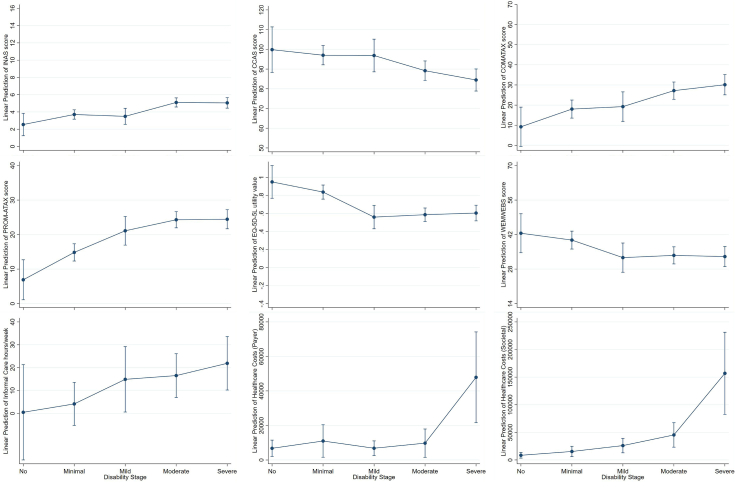
Margins plots with linear prediction of outcomes across disability stages **Footnotes:** Margins plots based on multivariate linear (patient-reported outcomes) and generalized estimating equation model preictions (economic outcomes; gamma family with log link) with random effects for study site (cluster) and adjusted for age, sex, education, disease duration. **Abbreviations:** CCAS, Cerebellar Cognitive Affective Syndrome Scale; FARS-ADL, Friedreich's Ataxia Rating Scale–Activities of Daily Living Subscale; WEMWBS, Warwick–Edinburgh Mental Well-being Scale; COMATAX, scale for the psychosocial impact of communication disabilities.

Fig. 2 shows the standardized coefficients using forest plots. Fig. 3 demonstrates the predicted trajectories for each clinical, patient-reported and health economic outcome using margin plots based on the regression models. Association of outcomes and SARA were comparable to associations with ADL stages (see Supplementary Figure S2 and Supplementary Table S5).

## Discussion

To our knowledge, PROFA represents the largest study to date on patient-reported, psychosocial, and health-economic outcomes in FA. This study highlights the significant impact of FA on patients' daily lives, both in terms of health and socioeconomic burden. As disability increased, patients experienced a worsening quality of life, reduced ability to manage everyday tasks, and increased communication difficulties. A higher need for both formal and informal care accompanied these changes. Notably, healthcare and societal costs rose sharply in later disease stages, especially among patients with full disability. These findings underscore the complex and progressive nature of FA and its wide-ranging effects on individuals, their families, and the healthcare system.

Our regression models confirmed distinct progression patterns across functional and psychosocial measures. The strong predictive value of both FARS-ADL and FARS disability stages suggests they capture similar aspects of functional decline, supporting the validity of FARS-ADL as a marker of disease severity. Given its reflection of clinically relevant thresholds of dependence, FARS-ADL shows promise as a complementary, remote, patient-reported outcome measure in both clinical and research contexts. Our study further supports the validity of the disease-specific PROM-Ataxia as a meaningful measure of HRQoL in Friedreich's ataxia (FA), showing a steady decline across disability stages with moderate effect sizes. This pattern likely reflects overlapping dimensions between PROM-Ataxia and clinical severity, particularly in terms of mobility and daily function. However, a ceiling effect at advanced stages suggests limited sensitivity in severe disability, warranting further research into its responsiveness to change and treatment effects. In contrast, generic HRQoL (EQ-5D-5L) and mental well-being (WEMWBS) scores showed a non-linear course. Both declined in early to moderate stages, but plateaued—or slightly improved—in later stages. EQ-5D-5L scores dropped from full health to moderate impairment, while WEMWBS scores remained consistently low, indicating a high psychological burden. These trends suggest that mental health in FA is not solely driven by physical disability but shaped by broader psychosocial factors and individual resilience. While we cannot exclude influencing factors such as resilience, adaptation, or scale limitations, this observation emphasizes the need for holistic care in FA that addresses both physical and psychological domains. Given the persistently low mental well-being across all stages, psychological support should be considered a priority throughout the disease course. Targeted interventions and improved access to mental health services may address one of the most frequently reported unmet needs in this population. Communication disabilities significantly increased across disease stages in our study. Given its social and emotional consequences, communication impairment should be addressed more effectively in FA management.^11^ COMATAX could be a valuable instrument for assessing communication disabilities in future research.

Regarding the utilization of healthcare services, late-onset patients were more likely to have children and receive more informal care. In contrast, formal care was more frequent among the typical-onset group. Informal care provided by close relatives was identified as the primary component of support and, thus, a cost driver in advanced FA disease stages, accounting for 58% of costs from a societal perspective and underscoring the burden on caregivers.^15^ The need for and reliance on informal care arise from various factors, such as complex care needs, financial constraints, and regional disparities in care provision. Still, the limited availability of formal services remains a major barrier.^29^ In this cross-sectional analysis, informal caregivers provided an average of 12.6 h of care per week, with time increasing by disease stage. However, only 16% reported reduced employment, suggesting that most care is provided alongside work. These numbers seem comparably low, compared to 26 h/week and 22%, as reported by Giunti and colleagues.^15^

Informal care time depends on the sample included, the methods used for assessment, and the inclusion or exclusion of activities of daily living and supervision tasks, making it difficult to compare these values across studies. If we only count the informal care hours provided by those who received care (58%), the mean informal care provision would be 21 h per week, which aligns with previous studies. However, the results of this and prior studies underline the burden on caregivers in FA.

Annual treatment costs for patients receiving care in specialized ataxia clinics are substantially higher compared to those without access to such services (€4788 vs €1638).^30^ The costs estimated in our study are consistent with those reported for patients treated in specialized settings and likely reflect better access to comprehensive care. Another study by Giunti et al. reported the total annual costs of FA in the UK, ranging from £11,818 to £18,774 per patient, highlighting the extensive burden on healthcare systems, caregivers, and society, as well as the potential health-economic role of slowing down disease progression.^15^

Intake of Omaveloxolone was more common among patients with a typical onset in our study. While we cannot rule out that this observation was biased by delayed access after its approval in 2024, this might also reflect the expectedly more rapid disease progression in these patients, as delineated in natural history studies.^8^^,^^9^ Consequently, a potential deceleration of disease progression is likely to result in a cumulative therapeutic effect of greater magnitude in these patients, which warrants investigation in future studies. Therefore, the impact on healthcare expenditures cannot be determined based on the findings of this cross-sectional study. Nonetheless, the longitudinal data may facilitate the estimation of precise values.

This study provides valuable insights into the progression of multiple patient-reported outcomes across disability stages in FA, a rare and complex disease. A key strength is the comprehensive assessment of various patient-reported outcomes within a well-characterized cohort. Additionally, PROFA's remote, app-based observational study design represents a significant methodological advancement, demonstrating the feasibility and potential of digital at-home assessments for decentralized clinical research. However, the analyses were limited by the relatively small sample size and potential selection bias, which resulted in an underrepresentation of patients with very low and very high disease severity. Some questionnaires were newly developed within this study, and others were never validated in FA, which limits their use in FA and the generalizability of the presented results. Therefore, evidence on the psychometric performance of these measures warrants further investigation.

Disease stages 0 and 6 were not represented in our cohort, which limits the generalizability of results on patients in these disability stages, and results from the models are, therefore, preliminary at this stage. However, recruitment is ongoing, and continuation of PROFA is planned until 2028. This upcoming data will allow for consideration of Omaveloxolone intake as an influencing factor, which may have impacted the outcomes, but has not been analyzed in our dataset to date. Despite being patient-centred, the use of FARS disability stages as the primary anchor point could impose a bias on the analyses, as the progression between the stages is largely defined by motor decline. Associations with the other outcome measures may therefore have influenced analyses and limited interpretability.

Additionally, our outcome analyses across disability stages did not account for the multisystemic aspects of FA, such as cardiomyopathy, diabetes and scoliosis, which may limit the generalisability of our results. Additionally, essential supportive aspects like rehabilitation were not included in our models, which may limit the interpretability of the results. Subgroup analyses were based on the more robust data on age of onset and disability stages, which is a limitation of this study.

The PROFA study highlights the multidimensional burden of FA, emphasizing that disease impact extends beyond motor impairment. Our findings underscore the critical need for holistic, multidisciplinary care approaches that address both physical and mental health needs, as well as support for informal caregivers who contribute substantially to patient care. Notably, the study reveals areas where healthcare provision may be insufficient. This information can be used to allocate limited healthcare resources more effectively, particularly concerning the more formal care services needed to meet individuals' needs and relieve the considerable strain placed on family caregivers providing informal care. Future research should focus on expanding longitudinal data collection to track patient-reported, psychosocial and health-economic outcomes trajectories more accurately and evaluate their responsiveness to therapeutic interventions, such as the recently approved FA medication Omaveloxolone.

## Contributors

***Conceptualization***: MGE, SB, FX, BM, TKlock, AD; ***Formal analysis***: BM, MGE, MB; ***Funding acquisition***: MGE, SB FX, BM, TKlock; ***Investigation***: CE, AH, MA, KF, VM, JF, SB, EI, KR, JBS, ATB, TKlop, LS, MM, DT, SS, RH, LP; ***Software***: EHD; ***Supervision***: MGE, SB, FX, BM, TKlock; ***Methodology***: MGE, SB, FX, BM, TKlock, BH, MB; ***Writing—original draft***: BM, MGE; ***Writing***—***review & editing***: SB, MB, SS, RH, LP, AI, BH, CE, AH, MA, KF, VM, JF, SB, EI, EHD, KR, JBS, AB, TKlop, LS, MM, DT, EHD, TKlock, AD, FX.

## Data sharing statement

The data supporting the results of this study are available on request from the corresponding author and the agreement of the PROFA consortium.

## Declaration of interests

**MGE** received research support from the National Ataxia Foundation and Ataxia UK, honoraria from Biogen, and travel support from Apario. All unrelated to this study. SB received consulting fees from Biogen. EI received honoraria from Biogen and Merz. **TK** received research support from Servier, UCB, Biogen, and the German Ministry of Research and Consulting Fees from Arrowhead, Bristol-Myer Squibb and UCB. **BM** received research support from the German Federal Joint Committee (G-BA), the German Ministry of Research, the Worch Foundation, the EuroQol Research Foundation and Biogen, and Consulting Fees from Biogen, the EuroQol Research Foundation and Lilly. **SB** received consulting fees from Biogen and Biohaven, honoraria from Ipsen, Abbvie, Merz Pharma and participated on advisoriy boards for Vico Therapeutics, Biogen and Biohaven. **LS** received Research support from PTC Therapeutics, and consulting fees from Reata Pharmaceuticals. **MM** was supported by the Deutsche Forschungsgemeinschaft (MI 709/2-1) and by the German Heredo Ataxia Society (DHAG) and received honoraria and travel expenses from Biogen, unrelated to this research. **CE** received honoraria from Biogen. **JS** received research support from Biogen, Lilly, and Eisai, consulting fees from Biogen, Reata, Lilly ans Eisai, honoraria from Biogen, Lilly and Eisai, and travel support from Lilly, Biogen, and Eisai. He is Speaker German Network of Memory Clinics and the Commission “Dementia” of the German Society of Neurology. **AD** received research support from the National Institute of Health (U01 NS104326), from ANR PROFA, ANR Cortex, Servier, Mavelife, Enroll (CHDI), Vico therapeutics, ASKIBO, and PTC therapeutics, and conculting fees from Biogen, UCB and HUNTIX. She participated in Boards for HUNTIX, Biogen and FRM, holds shares in patent B 06291873.5 and is president of the Société Francophone de Neurogénetique. **KR** revieved research support from Friedreich's Ataxia Research Alliance (FARA); Interdisciplinary Center for Clinical Research within the Faculty of Medicine at the RWTH Aachen University, Germany (OC2). **JF** was funded within the Advanced Clinician Scientist Programme (ACCENT, funded by the Federal Ministry of Research, Technology and Space (BMFTR) under the funding code (FKZ): 01EO2107) and as a PI of the iBehave Network, sponsored by the Ministry of Culture and Science of the State of North Rhine-Westphalia and received funding from the National Ataxia Foundation (NAF). She has received consultancy honoraria from Vico therapeutics and Biogen, travel support from NAF, FARA, Euro-Ataxia and AGI and participated in advisory boards for Vico. All other authors declare not conflicts of interest.
