# Supplementary material for: Patient-reported, psychosocial and health economic outcomes in mild to moderate Friedreich's ataxia: baseline results of the PROFA study
Source: Lancet Reg Health Eur. 2025 Dec 11;61:101552. doi: 10.1016/j.lanepe.2025.101552 (PMC12756708; doi:10.1016/j.lanepe.2025.101552)
Supplement: Supplementary Figures and Tables [file mmc1.docx]

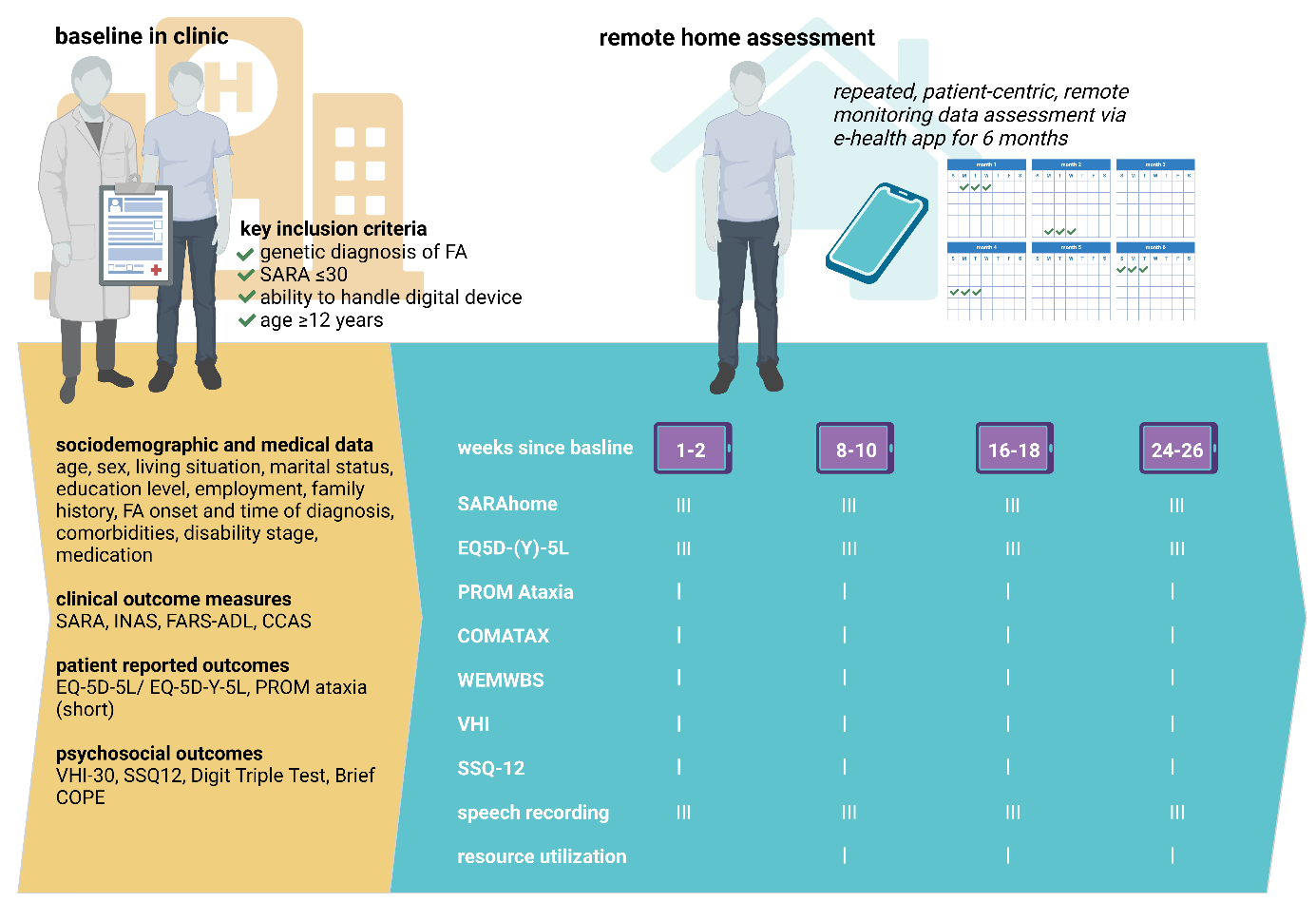


**Supplementary Figure 1**: Study design of the PROFA study. An on-site baseline visit is followed by 6 months of remote home assessments, which are submitted in blocks via an e-health app. SARAhome, EQ5D-(Y)-5L, and speech recordings are each performed on three consecutive days.

CCAS, Cerebellar Cognitive Affective Syndrome Scale; FARS-ADL, Friedreich's Ataxia Rating Scale - Activities of Daily Living Subscale; WEMWBS, Warwick-Edinburgh Mental Well-being Scale; COMATAX, scale for the psychosocial impact of communication disabilities; VHI, Voice Handicap Index; SSQ-12, Speech, Spatial and Qualities of Hearing Scale)

**
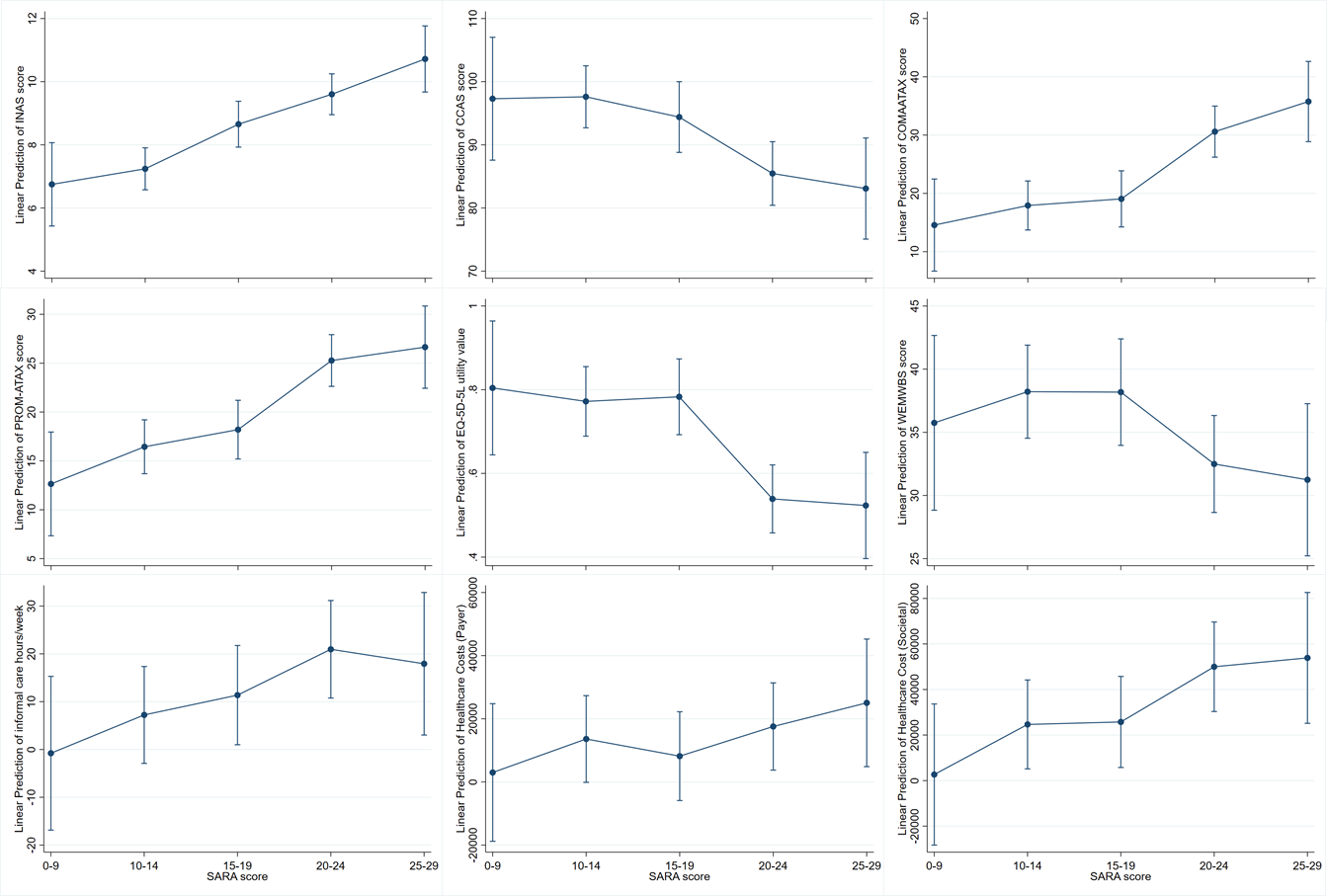
**

**Supplementary Figure 2:** Margins plots with linear prediction of outcomes across SARA categories

**Footnotes:** Margins plots based on multivariate linear (patient-reported outcomes) and generalized estimating equation models (economic outcomes; gamma family with log link) with random effects for study site (cluster) and adjusted for age, sex, education, and disease duration.

**Abbreviations:** CCAS, Cerebellar Cognitive Affective Syndrome Scale; FARS-ADL, Friedreich's Ataxia Rating Scale - Activities of Daily Living Subscale; WEMWBS, Warwick-Edinburgh Mental Well-being Scale; COMATAX, scale for the psychosocial impact of communication disabilities.

**Supplementary Table 1:** Description of outcome assessments of the PROFA study

| **Domain** | **Outcome measure** | **Description** |
| --- | --- | --- |
| **Assessment of clinical status/ severity of symptoms** | **SARA**  Scale for Assessment and Rating of Ataxia^1^ | consists of a neurologic examination of the core ataxia symptoms, summarized as ataxia severity on a scale ranging from 0-40, with higher values representing more severe ataxia. SARA is approved in FA and is commonly used to assess ataxia severity in research and routine clinical care settings.^2^ |
|  | **INAS**  Inventory of Non-Ataxia Signs^3^ | Captures additional neurological symptoms beyond ataxia, such as spasticity, neuropathy, and dystonia. The present symptoms are summed up to an INAS count, ranging from 0-16. |
|  | **CCAS**  Cerebellar Cognitive Affective Syndrome Scale^4^ | Assesses cognitive and affective dysfunctions related to cerebellar pathology, covering executive function, spatial cognition, and emotional regulation domains. The CCAS "raw score" (0-120 points) denotes the score achieved across all domains examined, with lower values indicating a poorer performance. The "pass/fail score" (values between 0-10 points) indicates the number of domains examined that were not passed. A score of 3 or more suggests a definite Cerebellar Cognitive Affective Syndrome diagnosis. |
|  | **FARS Disability Stages**^5^ | A clinical assessment of a patient's mobility status. The stages are as follows:  **0: Normal** (No symptoms); stage  **1:** minimal signs detected by the physician during screening (can run/jump without balance issues**, no disability**)  **2:** mild symptoms (balance issues, independent life, **minimal disability**)  **3:** moderate symptoms (needs support for walking, **mild disability**)  **4:** severe walking difficulty (requires walking aids, **moderate disability**)  **5:** wheelchair-bound (can use a wheelchair, **severe disability**)  **6:** total dependence (confined to bed/wheelchair, **total disability**).  The disability stage is this paper's main predictor of patient-reported, psychosocial, and economic health outcomes. |
| **Quality of life and mental well-being** | **FARS-ADL**^5^  Acivities of Daily Living Section of the Friedreich Ataxia Rating Scale^1^ | Evaluates the impact of disease progression on daily activities, including speech, swallowing, dressing, and mobility, with a score range between 0-36, with higher scores indicating greater functional impairment. |
|  | **EQ-5D-5L** and EQ-5D-Y-5L^6,7^ | The most widely used utility-based patient-reported outcome measure, covering five domains of Health Related Quality of Life (HRQoL) (i.e., mobility, self-care, usual activities, pain/discomfort, anxiety/depression) with five severity levels. The youth version (EQ-5D-Y-5L) consists of more child-appropriate wording used for participants aged 12 to 16 years. The German and French value sets were used to generate health utilities, ranging from one (full health) to -0.525 (worst health).^8,9^ |
|  | **PROM-Ataxia**  Patient Reported Outcome Measure– Ataxia^10^ | This study utilizes the short form of the PROM-Ataxia: A 10-item self-assessment of ataxia-related impairments, covering physical, mental health, and daily activities. It is based on the validated 70-item PROM-Ataxia questionnaire, which ranges from 0 to 40, with higher scores indicating higher impairment. |
|  | **WEMWBS**  Warwick-Edinburgh Mental Well-being Scale^11^ | A 14-item questionnaire measuring positive mental health, including optimism, relaxation, and social functioning, on a 5-point Likert scale, with higher scores (range 14-70) indicating better well-being. |
| **Communication disabilities** | **COMATAX** | A newly developed instrument to measure the psychosocial impact of communication disabilities. The self-rated questionnaire comprises 17 items, rated on a 5-point Likert scale (0-4). The development process was guided by suggestions from focus groups and individual interviews with individuals with ataxia, their caregivers, and professionals from various specialties. The topics covered involve speech, hearing, language, emotions, the connection between communication and fatigue, and the psychological implications of communication difficulties. The 17 items relate to the following domains: Item 1: difficult articulation, Item 2: slow speech, Item 3: weak voice, Item 4: shortness of speaking breath, Item 5: gives up long sentences, Item 6: group discussions too fast, Item 7: difficulty in finding words, Item 8: difficulty in dual task with speech, Item 9: speech more difficult when tired, Item 10: understanding difficult in noise, Item 11: hearing difficult in quiet, Item 12: written communication difficult, Item 13: emotional reactions different from others, Item 14: emotions felt more strongly, Item 15: friendships difficult, Item 16: trouble accepting my speech, Item 17: tiring communication, Item 18: Most bothersome symptom of the 17 privously assessed domains. Scores range from 0 to 68. Higher scores indicate a more severe impact on communication difficulties. Validation and normalization are ongoing in the PROFA project. |
|  | **Triplet Digit Noise Test**^12^ | An Assessment of Hearing Loss. The patient listens to digits produced in adaptive noise. Difficulty rises with every correct answer, as the noise increases and the voice becomes less intelligible. Patients are classified into three categories: no hearing impairment, low probability of hearing impairment, and high probability of hearing impairment. |
| **Health resource utilization** | **Health-Related Resource Use (FIMA) and Resource Utilization questionnaires (RUD)**^13,14^ | Assesses healthcare utilization during the past two months. Healthcare utilization includes physician visits, in-hospital treatments, medication, therapies, medical aids and formal home and institutionalized care. Informal care encompasses caregiver support for daily living activities, instrumental daily living activities, and the short-term and long-term productivity losses of caregivers. |
| *References:*  *1 Schmitz-Hübsch T, Du Montcel ST, Baliko L, et al. Scale for the assessment and rating of ataxia: development of a new clinical scale. Neurology 2006; 66: 1717–20.* [*https://doi.org/10.1212/01.wnl.0000219042.60538.92*](https://doi.org/10.1212/01.wnl.0000219042.60538.92%20)  *2 Bürk K, Mälzig U, Wolf S, et al. Comparison of three clinical rating scales in Friedreich ataxia (FRDA). Movement disorders: official journal of the Movement Disorder Society 2009; 24: 1779–84. https://doi.org/10.1002/mds.22660*  *3 Jacobi H, Rakowicz M, Rola R, et al. Inventory of Non-Ataxia Signs (INAS): validation of a new clinical assessment instrument. Cerebellum (London, England) 2013; 12: 418–28. https://doi.org/10.1007/s12311-012-0421-3*  *4 Hoche F, Guell X, Vangel MG, Sherman JC, Schmahmann JD. The cerebellar cognitive affective/Schmahmann syndrome scale. Brain: a journal of neurology 2018; 141: 248–70. https://doi.org/10.1093/brain/awx317*  *5 Subramony SH, May W, Lynch D, et al. Measuring Friedreich ataxia: Interrater reliability of a neurologic rating scale. Neurology 2005; 64: 1261–62. https://doi.org/10.1212/01.WNL.0000156802.15466.79*  *6 Herdman M, Gudex C, Lloyd A, et al. Development and preliminary testing of the new five-level version of EQ-5D (EQ-5D-5L). Qual Life Res 2011; 20: 1727–36. https://doi.org/10.1007/s11136-011-9903-x*  *7 Kreimeier S, Åström M, Burström K, et al. EQ-5D-Y-5L: developing a revised EQ-5D-Y with increased response categories. Qual Life Res 2019; 28: 1951–61. https://doi.org/10.1007/s11136-019-02115-x*  *8 Ludwig K, Graf von der Schulenburg J-M, Greiner W. German Value Set for the EQ-5D-5L. Pharmacoeconomics 2018; 36: 663–74. https://doi.org/10.1007/s40273-018-0615-8*  *9 Andrade LF, Ludwig K, Goni JMR, Oppe M, Pouvourville G de. A French Value Set for the EQ-5D-5L. Pharmacoeconomics 2020; 38: 413–25. https://doi.org/10.1007/s40273-019-00876-4*  *10 Schmahmann JD, Pierce S, MacMore J, L'Italien GJ. Development and Validation of a Patient-Reported Outcome Measure of Ataxia. Movement disorders: official journal of the Movement Disorder Society 2021; 36: 2367–77. https://doi.org/10.1002/mds.28670*  *11 Tennant R, Hiller L, Fishwick R, et al. The Warwick-Edinburgh Mental Well-being Scale (WEMWBS): development and UK validation. Health and quality of life outcomes 2007; 5: 63. https://doi.org/10.1186/1477-7525-5-63*  *12 Lunney M, Wiebe N, Howarth T, et al. Performance of Hearing Test Software Applications to Detect Hearing Loss. JAMA Netw Open 2025; 8: e252166. https://doi.org/10.1001/jamanetworkopen.2025.2166*  *13 Seidl H, Bowles D, Bock J-O, et al. FIMA--Fragebogen zur Erhebung von Gesundheitsleistungen im Alter: Entwicklung und Pilotstudie. Gesundheitswesen (Bundesverband der Arzte des Offentlichen Gesundheitsdienstes (Germany)) 2015; 77: 46–52. https://doi.org/10.1055/s-0034-1372618*  *14 Wimo A, Gustavsson A, Jönsson L, Winblad B, Hsu M-A, Gannon B. Application of Resource Utilization in Dementia (RUD) instrument in a global setting. Alzheimers Dement 2013; 9: 429-435.e17.* [*https://doi.org/10.1016/j.jalz.2012.06.008*](https://doi.org/10.1016/j.jalz.2012.06.008) | | |

**Supplementary Table 2:** Unit costs used to monetarize healthcare service utilization

| Perspectives | | Cost sector | Services | Units | Unit cost & source for monetary valuation |
| --- | --- | --- | --- | --- | --- |
| SOCIETAL PERSPECIVES | **PAYER PERSPECTIVE** | **Medical care** |  |  |  |
|  |  | Physician treatment | GP or specialists | Contact | Cost per contact |
|  |  | In-patient treatment | In-hospital treatment and rehabilitation | Days | Average per diem cost for in-hospital treatment & rehabilitation |
|  |  | Medications | Regularly prescribed (Rx-drugs) and Over-the-Counter (OTC) drugs | Quantity | Pharmaceutical Index of the Scientific Institute of the AOK |
|  |  | Medical aids | Aids like tub-lift, tub-seats, walking sticks, walkers and others | Quantity | Market prices |
|  |  | Therapies | Occupational therapy, speech therapy, physiotherapy and others | Contacts | Cost per contact and reimbursement schedules of statutory health insurance |
|  |  | **Formal care** |  |  |  |
|  |  | Nursing home care | Short-term care (e.g., partial day- and night-time nursing care) and long-term care | Hours | Average gross wage plus non-wage labor cost of all employees |
|  |  | Ambulatory care | Home care provided by professionals | Hours | Average gross wage plus non-wage labor cost of all employees |
|  | **FAMILY PERSPECTIVE** | **Informal care** | Caregivers time spent regarding ADL (e.g., body care, assistance on the toilet, eating, dressing, mobilization) and IADL (e.g., assistance for telephone use, shopping, cooking, washing laundry, cleaning the household, medication intake) | Hours | Opportunity cost approach: a) Opportunity cost of lost production was valued using the average gross wage plus non-wage labor cost of all employees (25.46 €)*. Hours spent for informal care are limited to a maximum of 18 hours per day. In case that the caregiver is working or finally quit work due to the provision of informal care, informal care time is reduced by caregivers lost production time. |

******Muntendorf LK, Brettschneider C, Konnopka A, König HH. Updating standardized unit costs from a societal perspective for health economic evaluation. Gesundheitswesen. 2024 May;86(5):389-393. German. doi: 10.1055/a-2169-1480.*

**Supplementary Table 3:** Description of missing values

|  | **Missing values**  n (%) |
| --- | --- |
| **Sociodemographic** |  |
| Age | 0 (0.0) |
| Sex | 0 (0.0) |
| Having children | 1 (1.0) |
| Education level | 0 (0.0) |
| Employment | 0 (0.0) |
| **Clinical characteristics** |  |
| Age of onset | 0 (0.0) |
| FA duration | 0 (0.0) |
| Ataxia severity (SARA) | 0 (0.0) |
| FA Disability stage | 1 (1.0) |
| Short allele (FXN GAA1) | 15 (14.9) |
| Long allele (GAA2) | 22 (21.8) |
| Combined (GAA1&2/2) | 22 (21.8) |
| INAS | 0 (0.0) |
| CCAS | 6 (5.9) |
| Hearing problems | 4 (4.0) |
| **Patient-reported outcomes** |  |
| EQ-5D-5L | 3 (3.0) |
| PROM-Ataxia | 2 (2.0) |
| FARS-ADL | 0 (0.0) |
| WEMWBS | 15 (14.9) |
| COMATAX | 15 (14.9) |
| **Health service utilization** |  |
| Social care access^**^ | 32 (31.7) |
| Hospitalized^*^ | 32 (31.7) |
| Formal care support^*^ | 32 (31.7) |
| Omaveloxolone intake | 0 (0.0) |
| **Informal care** |  |
| Informal care provision^*^ | 32 (31.7) |
| Hours per week | 32 (31.7) |
| Caregiver employment reduction | 34 (33.7) |
| **Health-care costs in € values** |  |
| Payer perspective | 32 (31.7) |
| Societal perspective | 32 (31.7) |

**Abbreviations:** FA, Friedreich Ataxia; SD, standard deviation. CCAS, Cerebellar Cognitive Affective Syndrome Scale; FARS-ADL, Friedreich's Ataxia Rating Scale - Activities of Daily Living Subscale; WEMWBS, Warwick-Edinburgh Mental Well-being Scale; COMATAX, scale for the psychosocial impact of communication disabilities; Hearing

**Footnotes:** ^*^ 5 patients with a mutation point in our population, ^**^ during the last 2 months

**Supplementary Table 4:** Recruited participants per study center

|  | **Aachen** | **Bonn** | **München** | **Tübingen** | **Innsbruck** | **Paris** |
| --- | --- | --- | --- | --- | --- | --- |
| **Participants**, n (%) | 16 (15.7) | 11 (10.8) | 7 (6.9) | 7 (6.9) | 11 (10.8) | 50 (49.0) |
| **Age**, mean (SE) | 26.8 (2.9) | 35.6 (4.2) | 36.0 (7.9) | 31.7 (9.4) | 41.4 (4.2) | 36.4 (1.9) |
| **Age of onset**, mean (SE) | 14.9 (1.7) | 20.5 (4.2) | 19.6 (6.1) | 20.0 (3.4) | 21.8 (3.3) | 19.6 (1.5) |
| **Disease duration (years)**, mean (SE) | 11.9 (2.2) | 15.1 (2.0) | 16.4 (3.4) | 11.7 (2.2) | 19.5 (2.9) | 16.8 (1.2) |
| **Disability stage**, median | 3.5 | 6 | 4 | 3 | 3.5 | 5 |
| **SARA score**, mean (SE) | 14.9 (1.5) | 20.5 (1.7) | 13.6 (1.4) | 13.6 (1.1) | 16.8 (1.7) | 19.0 (0.8) |

**Abbreviations:** SARA, Scale for the assessment and rating of ataxia; SE, standard error; n, number.

**Supplementary Table 5:** Association between standardized outcomes and SARA categories (SARA scores 0-9, 10-14. 15-19, 20-24, and 25-29)

| **Standardized Outcomes** | Estimates (beta coef.) | Standard Error | 95% | CI | p-value | R^2^ | R^2^ gain |
| --- | --- | --- | --- | --- | --- | --- | --- |
| CCAS^1^ | -0.35 | 0.09 | -0.53 | -.16 | **0.000** | 17.4 | 12.7 |
| EQ-5D-5L^2^ | -0.39 | 0.09 | -0.57 | -.21 | 0.**000** | 22.3 | 15.8 |
| PROM-Ataxia^3^ | 0.47 | 0.08 | 0.30 | .63 | **0.000** | 34.2 | 22.6 |
| FARS-ADL^4^ | 0.65 | 0.07 | 0.51 | .78 | **0.000** | 54.4 | 43.5 |
| WEMWBS^5^ | -0.21 | 0.10 | -0.42 | -.01 | **0.041** | 9.4 | 4.7 |
| COMATAX^6^ | 0.48 | 0.09 | 0.30 | .66 | **0.000** | 34.4 | 23.8 |
| Informal care time^7^ | 0.25 | 0.11 | 0.03 | .46 | **0.029** | 16.9 | 6.4 |
| Health-care Costs (Payer)^8^ | 0.16 | 0.12 | -0.07 | .39 | 0.171 | 10.5 | 2.6 |
| Health-care Costs (Societal)^9^ | 0.30 | 0.11 | 0.08 | .51 | **0.006** | 21.1 | 9.4 |

**Abbreviations:** CI, confidence interval; R^2^, coefficient of determination; CCAS, Cerebellar Cognitive Affective Syndrome Scale; FARS-ADL, Friedreich's Ataxia Rating Scale - Activities of Daily Living Subscale; WEMWBS, Warwick-Edinburgh Mental Well-being Scale; COMATAX, scale for the psychosocial impact of communication disabilities.

**Footnotes:** Linear regression model with random effects for study site (cluster) and adjusted for age, sex, education, disease duration; bold numbers indicate a statistically significant difference (p≤0.05); ^1^ n=95; 6 clusters, 15.8 observation per cluster, Wald chi^2^(5)=18.80, p=0.0021; ^2^ n=97; 6 clusters, 16.2 observation per cluster, Wald chi^2^(5)=26.16, p=0.0001; ^3^ n=98; 6 clusters, 16.3 observation per cluster, Wald chi^2^(5)=47.73, p=0.0000; ^4^ n=101; 6 clusters, 16.8 observation per cluster, Wald chi^2^(5)=113.30, p=0.0000; ^5^ n=85; 6 clusters, 14.2 observation per cluster, Wald chi^2^(5)=8.23, p=0.1441; ^6^ n=84; 6 clusters, 14.0 observation per cluster, Wald chi^2^(5)=31.47, p=0.0000; ^7^ n=66; 6 clusters, 11.0 observation per cluster, Wald chi^2^(5)=15.74, p=0.0076; ^8^ n=69; 6 clusters, 11.5 observation per cluster, Wald chi^2^(5)=7.41, p=0.1920; ^9^ n=696; 6 clusters, 11.5 observation per cluster, Wald chi^2^(5)=16.86, p=0.004
